# Neural development in the tardigrade *Hypsibius dujardini* based on anti-acetylated α-tubulin immunolabeling

**DOI:** 10.1186/s13227-015-0008-4

**Published:** 2015-04-25

**Authors:** Vladimir Gross, Georg Mayer

**Affiliations:** Animal Evolution and Development, Institute of Biology, University of Leipzig, Talstraße 33, 04103 Leipzig, Germany; Department of Zoology, Institute of Biology, University of Kassel, Heinrich-Plett-Str. 40, D-34132 Kassel, Germany

**Keywords:** Axonogenesis, Nervous system, Panarthropoda, Segmental ganglia, Tardigrada, Water bears

## Abstract

**Background:**

The tardigrades (water bears) are a cosmopolitan group of microscopic ecdysozoans found in a variety of aquatic and temporarily wet environments. They are members of the Panarthropoda (Tardigrada + Onychophora + Arthropoda), although their exact position within this group remains contested. Studies of embryonic development in tardigrades have been scarce and have yielded contradictory data. Therefore, we investigated the development of the nervous system in embryos of the tardigrade *Hypsibius dujardini* using immunohistochemical techniques in conjunction with confocal laser scanning microscopy in an effort to gain insight into the evolution of the nervous system in panarthropods.

**Results:**

An antiserum against acetylated α-tubulin was used to visualize the axonal processes and general neuroanatomy in whole-mount embryos of the eutardigrade *H. dujardini*. Our data reveal that the tardigrade nervous system develops in an anterior-to-posterior gradient, beginning with the neural structures of the head. The brain develops as a dorsal, bilaterally symmetric structure and contains a single developing central neuropil. The stomodeal nervous system develops separately and includes at least four separate, ring-like commissures. A circumbuccal nerve ring arises late in development and innervates the circumoral sensory field. The segmental trunk ganglia likewise arise from anterior to posterior and establish links with each other via individual pioneering axons. Each hemiganglion is associated with a number of peripheral nerves, including a pair of leg nerves and a branched, dorsolateral nerve.

**Conclusions:**

The revealed pattern of brain development supports a single-segmented brain in tardigrades and challenges previous assignments of homology between tardigrade brain lobes and arthropod brain segments. Likewise, the tardigrade circumbuccal nerve ring cannot be homologized with the arthropod ‘circumoral’ nerve ring, suggesting that this structure is unique to tardigrades. Finally, we propose that the segmental ganglia of tardigrades and arthropods are homologous and, based on these data, favor a hypothesis that supports tardigrades as the sister group of arthropods.

**Electronic supplementary material:**

The online version of this article (doi:10.1186/s13227-015-0008-4) contains supplementary material, which is available to authorized users.

## Background

Tardigrades are microscopic invertebrates that are found worldwide in a variety of marine and freshwater environments as well as in lichens and cushion plants [1]. They form a monophyletic group characterized by a number of features such as five body segments, four pairs of clawed legs, and a buccopharyngeal apparatus [2]. The position of tardigrades remains controversial, as they display a number of cycloneuralian features (for example, terminal mouth and triradiate pharynx) [3,4], while other characters typical of panarthropods may have been lost as a result of miniaturization [5]. More ambiguity comes from several molecular analyses that place tardigrades with nematodes [6-9], although this may be largely due to a long-branch attraction artifact [10,11]. Despite these exceptions, they are widely considered to be members of the Panarthropoda (Tardigrada + Onychophora + Arthropoda), although the relationship between the three groups remains poorly resolved [10-13].

One potentially useful technique for clarifying this issue is the study of the morphology and development of the nervous system, as such previous investigations have already contributed valuable insights to our understanding of panarthropod evolution [14-19]. However, missing or conflicting data from several key groups present an obstacle for this field. Such is the case regarding the Tardigrada, where previous investigations of embryonic development have yielded contradictory results, in particular regarding the nervous system. For example, Marcus [20] and Eibye-Jacobsen [21] both described a single ventral neural anlage that later segments to form all components of the nervous system concurrently, including the brain. Hejnol and Schnabel [22], on the other hand, used 4D microscopy to trace the ganglia back to individual neural progenitor cells, finding no evidence for a unitary neural structure at any point during development. Opposing views also represent several other aspects of tardigrade neuroanatomy, for example, the number of brain segments [23-26], the structure of the circumbuccal nerve ring [11,24,26], and homologies of the segmental ganglia [11,26,27].

On the other hand, some features of tardigrade neuroanatomy are undeniably similar to those of the arthropods, the most striking being the presence of a segmental, ‘rope ladder-like’ nervous system [11,24,28,29]. This is in contrast to the onychophorans, which lack ganglia but instead have a pair of ventral nerve cords that exhibit a medullary organization and are linked by numerous median commissures in a non-segmental fashion [30]. Thus, the use of onychophorans for comparative purposes is limited in this sense despite the fact that their nervous system, including its origin and development, has been detailed comprehensively [15-17,31-37]. Unfortunately, adequate morphological data on tardigrade organ systems, such as the nervous system, are almost exclusively restricted to adults [11,23-25,28,29,38-41], with studies on embryos being scarce and, in some cases, controversial [20-22,42-44].

**Figure 1:**
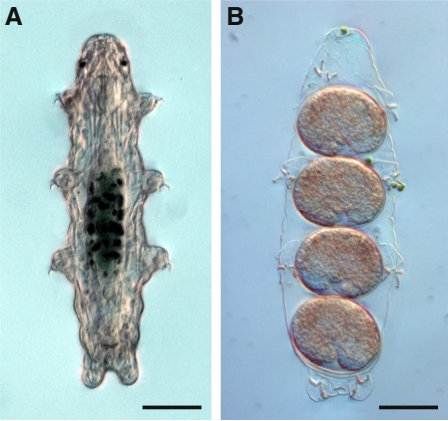
Habitus of an adult specimen and deposited eggs with embryos of the eutardigrade *Hypsibius dujardini*. Differential interference contrast (DIC) light micrographs. Anterior is up. **(A)** A live, adult specimen in dorsal view. The gut full of ingested algae appears as a dark green region in the midbody. **(B)** A shed cuticle containing four eggs with embryos. Notice the curled shape of each embryo. Scale bars: (A), (B), 50 μm.

Thus, these issues warrant a detailed investigation of tardigrade neural development using modern techniques, such as immunolabeling in conjunction with confocal laser scanning microscopy (CLSM). Herein, we use an antiserum directed against acetylated α-tubulin - a major component of axonal processes - as a general marker of neural structures to examine the mode of neural development in the emerging model tardigrade species *Hypsibius dujardini* (Figure 1A, B) [42,45] and compare it to that of the other panarthropods.

## Methods

### Specimen culture and collection of embryos

Specimens of *Hypsibius dujardini* (Doyère, 1840) (Eutardigrada, Hypsibiidae) were purchased from Sciento (Manchester, United Kingdom) and cultured at room temperature (20°C to 24°C) in Petri dishes in mineral water (Volvic, Danone Waters Deutschland GmbH, Frankfurt am Main, Germany) containing algae of a unicellular *Clorococcum* species (Sciento). The water and algae were replaced every 10 days. Populations were subcultured intermittently in new Petri dishes. Molted exuvia containing eggs were collected using a glass micropipette and cut with electrolytically sharpened tungsten needles to release the eggs. Approximately 200 to 400 embryos were collected for each experiment. Staging and developmental time estimates, based on nuclear labeling, are according to Gabriel *et al*. [42].

### Immunohistochemistry

The following protocol for embryo preparation is based on the one used by Gabriel and Goldstein [46]. Embryos were collected in 1 ml PBT (5 mM phosphate-buffered saline, pH 7.4, plus 1% Triton X-100) and subsequently incubated in a solution containing chitinase (50 mg/ml; Sigma-Aldrich, St. Louis, MO, USA) and chymotrypsin (15 mg/ml; Sigma-Aldrich, St. Louis, MO, USA) in PBT for 1 h at room temperature. After 3 × 5 min washes in PBT, embryos were dehydrated in 1 ml chilled (−20°C), absolute methanol at 4°C for 20 min, then run through a methanol series (5 min each in 90%, 70%, 50% methanol). Fixation was done using 1 ml of 4% paraformaldehyde in PBT for 10 min at room temperature followed by 15 min at 4°C. The embryos were then washed 4 × 5 min in PBT and left therein overnight. On the following day, the embryos were manually dissected from the chorion using tungsten needles and rinsed briefly in PBT. Blocking to prevent unspecific antibody binding was done using 10% normal goat serum (NGS) in PBT for 1 h at room temperature. Incubation with the primary antibody (mouse anti-acetylated α-tubulin; Sigma-Aldrich, St. Louis, MO, USA; diluted 1:1,000 in PBT, plus 1% NGS) was done overnight at room temperature on a slow shaker. The specimens were then washed 3 × 5 min and 2 × 20 min, followed by a buffer change every 1 to 2 h for the rest of the day. In the evening, the embryos were transferred to a solution containing the secondary antibody (Alexa Fluor® 568 goat anti-mouse IgG; Invitrogen, Carlsbad, CA, USA; diluted 1:1,000 in PBT plus 1% NGS) and incubated overnight at room temperature on the shaker. On the following day, the embryos were washed 2 × 5 min, 2 × 15 min, and 2 × 30 min in PBT, incubated in SYBR® Green (Life Technologies, Carlsbad, CA, USA) for 2 h, mounted in ProLong Gold (Molecular Probes®, Eugene, OR, USA) between two glass coverslips, and left to cure in the dark at room temperature. After 48 h, the slides were sealed with nail polish to prevent oxidation.

### Data acquisition and image processing

For live imaging, adult tardigrades were anesthetized by asphyxiation with carbonated water for at least 4 h and mounted in distilled water on glass microscope slides. The specimens were imaged using a Leica Leitz DMR compound light microscope (Leica Microsystems, Wetzlar, Germany) equipped with a color digital camera (PCO AG SensiCam, Kelheim, Germany). Multiple image planes were fused into a focused projection using Adobe (San Jose, CA, USA) Photoshop CS6. A total of 64 fluorescently labeled, whole-mount embryos were scanned using a Leica TCS STED confocal laser scanning microscope (Leica Microsystems, Wetzlar, Germany) and the resulting z-stacks were analyzed using ImageJ v1.48 [47] and Imaris v7.2.1 (Bitplane, South Windsor, CT, USA). Final assembly and labeling of figures was done using Adobe Illustrator CS6.

## Results

### Neural development in the head

**Figure 2:**
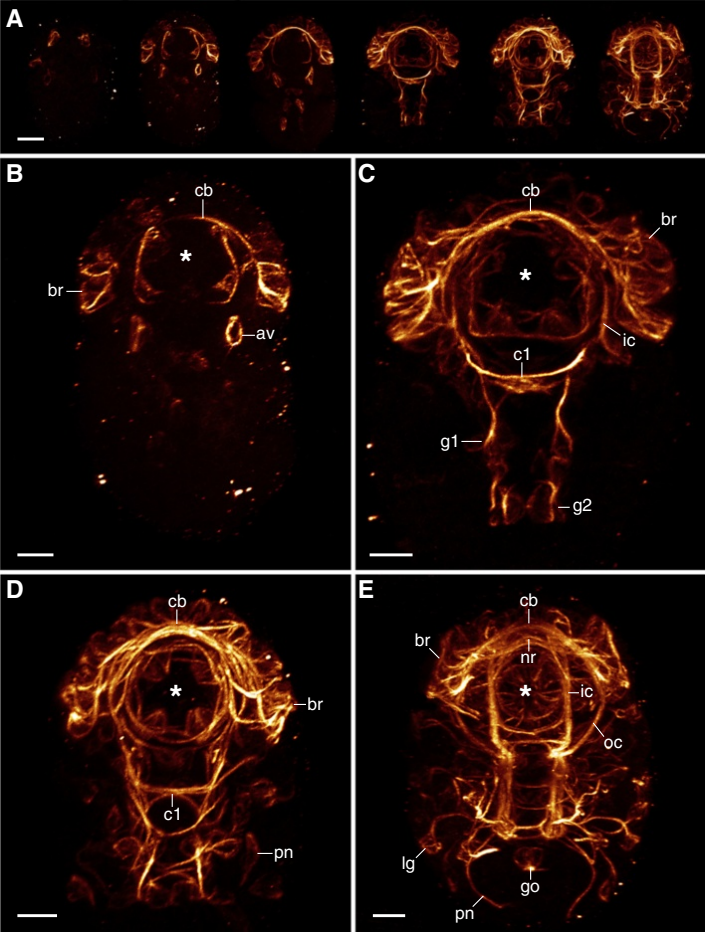
Neural development in embryos of *Hypsibius dujardini* in ventral view*.* Maximum CLSM projections of embryos of successive developmental stages labeled for acetylated α-tubulin. Asterisks indicate the position of the future mouth. **(A)** Overview of development from earliest (left) to latest (right) stages analyzed. Note that the nervous system develops from anterior to posterior. **(B-E)** Details of subsequent developmental stages. Note that the anteroventral cells arise early in development (in (B)) and that the inner connectives are formed prior to the outer connectives (in (C)). Peripheral neurons arise only after the central nervous system has been established (in (D)). **(E)** A nearly complete embryo with the developing anlage of the gonad already present. Abbreviations: av, anteroventral cells; br, brain cells; c1, central commissure of the first trunk ganglion; cb, developing central brain neuropil (arising from a single commissure); g1, g2, anlagen of the first and second trunk ganglia; go, gonad anlage; ic, inner connectives; lg, leg ganglion; nr, circumbuccal nerve ring; oc, outer connective; pn, peripheral neuron. Scale bars: (A), 10 μm; (B-E), 5 μm.

**Figure 3:**
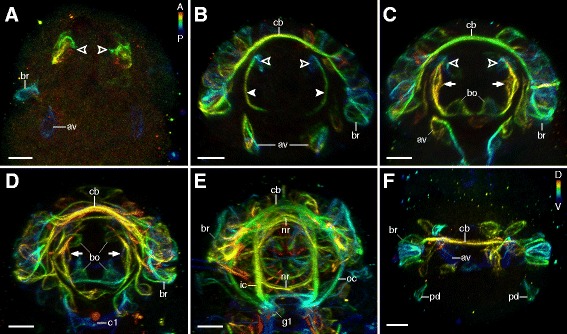
Details of neural development in the head region of *Hypsibius dujardini*. Maximum CLSM projections of embryos labeled for acetylated α-tubulin and depth-coded along the anteroposterior (in (A-E); orientation of depth-coding in (A)) or dorsoventral axis (in (F)). Dorsal is up in (A-E); anterior is up in (F). Hollow arrowheads indicate the first neurons of the stomodeal nervous system. Solid arrowheads demarcate stomodeal axons. Arrows point to neurons of the first stomodeal commissure. **(A)** Early-stage embryo showing the first three groups of neural cells. **(B-E)** Series of subsequent embryonic stages. Note that the first stomodeal neurons are posterior to the first brain commissure (=future central brain neuropil) but project axons anteriorly (in (B)). The ventral pair of buccal sensory organs arises before the dorsal pair (in (C), (D)). Also note that the neurons of the first stomodeal commissure are anterior to the brain and central brain neuropil (in (C)). **(E)** Late-stage embryo showing a complete circumbuccal nerve ring. **(F)** Dorsal view of the brain in an embryo of the same developmental stage as in (B). Notice the two pairs of posterodorsal cells (extracerebral neurons *sensu* ref. [23]) lying outside the anlage of the brain. Abbreviations: A, anterior; av, anteroventral cells; bo, buccal sensory organs; br, brain cells; c1, central commissure of the first trunk ganglion; cb, developing central brain neuropil (arising from a single commissure); D, dorsal; g1, first trunk ganglion anlage; ic, inner connective; nr, circumbuccal nerve ring; oc, outer connective; P, posterior; pd, posterodorsal cells; V, ventral. Scale bars: (A-F), 5 μm.

**Figure 4:**
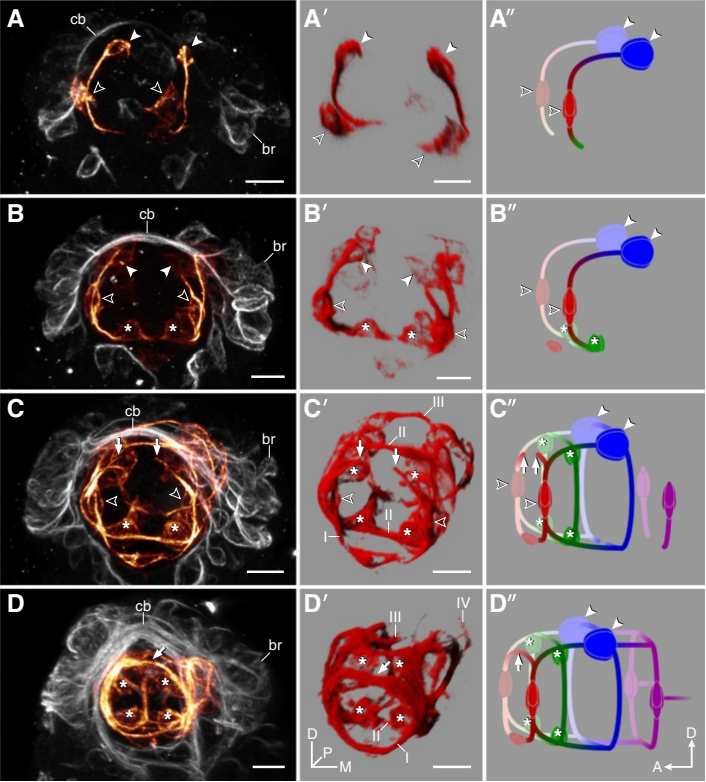
Development of the stomodeal nervous system in *Hypsibius dujardini*. Developmental series from earliest (A) to latest (D) embryonic stages. **(A-D)** CLSM projections showing the stomodeal nervous system (selectively colored in orange). Orientation as in (D′). Anti-acetylated α-tubulin immunolabeling. **(A**′-**D**′**)** Volume rendering of the corresponding structures. **(A**″-**D**″**)** Diagrammatic representation of the stomodeal nervous system in lateral view. Stomodeal commissures are numbered (I to IV). Note that neurons of the third stomodeal commissure (solid arrowheads) arise first (A) and project axons toward cells of the first commissure (hollow arrowheads), which only closes later in development (arrows in (C-C″), (D-D″)). The buccal sensory organs (asterisks in (B-B″), (C-C″), (D-D″)) contribute to the second commissure. Abbreviations: br, brain cells; cb, developing central brain neuropil (arising from a single commissure); D, dorsal; M, median; D, dorsal. Scale bars: (A-D), (A′-D′), 5 μm.

**Figure 5:**
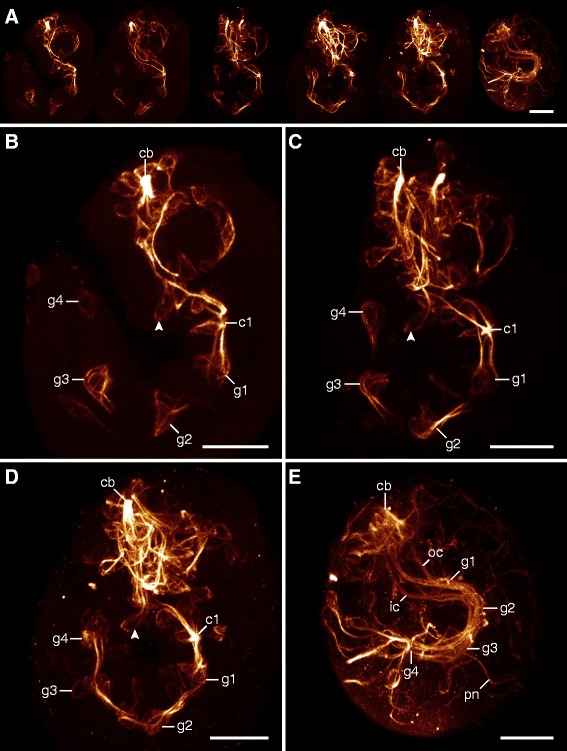
Neural development in embryos of *Hypsibius dujardini* in lateral view. Maximum CLSM projections of embryos labeled for acetylated α-tubulin. Anterior is up in all images. **(A)** Overview of successive embryonic stages demonstrating the development of the central nervous system in the trunk. **(B-E)** Detail of early (in (B)) to late embryonic stages (in (E)). Arrowheads indicate the position of the anteroventral cells in the head region. Abbreviations: cb, developing central brain neuropil; c1, central commissure of the first trunk ganglion; g1 to g4, anlagen of trunk ganglia 1 to 4; ic, inner connective; oc, outer connective; pn, peripheral nerve. Scale bars: (A-E), 10 μm.

**Figure 6:**
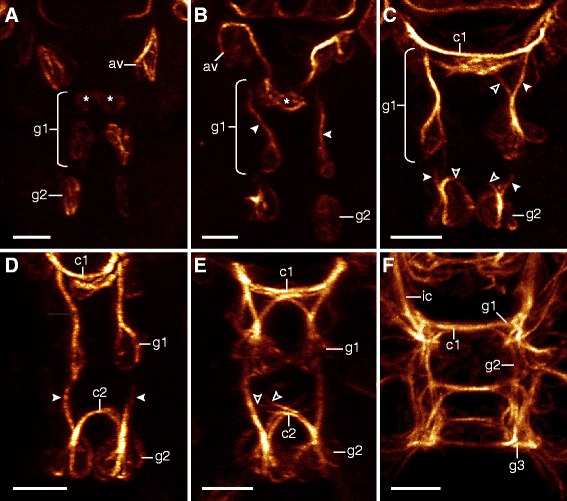
Development of the anterior two trunk ganglia in *Hypsibius dujardini*. Confocal z-series micrographs of embryos labeled for acetylated α-tubulin in ventral view. Anterior is up in all images. Contralateral and longitudinal pioneering axons are indicated by hollow and solid arrowheads, respectively. Asterisks (in (A) and (B)) indicate cells that will provide fibers to the central commissure of the first trunk ganglion. **(A**-**F)** Embryos of subsequent developmental stages. Each hemiganglion projects one longitudinal and one commissural pioneering axon (in (C)). Later in development, contralateral commissures grow toward the anterior adjacent segment (in (E)). Abbreviations: av, anteroventral cells; c1, c2, commissures of trunk ganglia 1 and 2; g1 to g3, anlagen of trunk ganglia 1 to 3; ic, inner connectives. Scale bars: (A-F), 5 μm.

Axonogenesis in *H. dujardini* proceeds rapidly, beginning approximately 40 h after egg deposition and finishing 5 to 10 h later (stage 16, *sensu* Gabriel *et al.* [42]; Additional file 1). The first neurons arise in the head and are associated with the brain and stomodeal complex (Figures 2A, 3A, and 4A). The brain cells arise dorsolaterally as clusters of neurons, which project their axons contralaterally over the stomodeal complex (Figures 2A, B and 3A, B). As the dorsal, saddle-like brain continues to develop, more cells arise from dorsolateral to dorsal along the thickening central commissure of the brain (=anlage of the central brain neuropil) (Figures 2A, B, C, D, E and 3A, B, C, D, E). Although neurons associated with the brain lie at various depths with respect to the body surface (Additional file 2), they are all positioned dorsally or dorsolaterally relative to the central brain commissure throughout development (Figures 3A, B, C, D and 4A, B, C, D). On the ventral side of the head, a pair of anteroventral (AV) cells arises early in development (Figure 3A). These cells become connected by posteriorly growing axons to the central commissure of the first trunk ganglion, followed by anteriorly growing axons that enter the circumbuccal nerve ring (Figures 2B, 3A, B, C, 5B, C, D, and 6A, B). The posteriorly oriented axons represent the first intersegmental neural tracts in the embryo. Later in development, the AV cells also grow dendritic neurites toward the body surface (Additional file 3). In addition, there are two pairs of posterodorsal cells that send their fibers into the dorsolateral brain region (Figure 3F). These cells are extracerebral neurons that lie within the epidermal cell layer, separate from the brain anlage.

The stomodeal nervous system of *H. dujardini* is interconnected by four ring-like commissures that, in contrast to the general pattern of neural development, do not develop in an anterior-to-posterior progression, as the third ring-like commissure develops first, followed by the second, first, and fourth commissures (Figure 4A, B, C, D, A′, B′, C′, D′, A″, B″, C″, D″). Neural development of the stomodeal nervous system begins with a pair of stomodeal cell clusters that are associated with the future third stomodeal commissure (Figures 3A and 4A, A′, A″). These cells arise concurrently with the initial brain cells and AV cells (Figure 3A) and are located posteroventral to the future developing central brain neuropil (Figure 3B). They also send fibers both anteriorly, to cells of the future first commissure, as well as posteriorly, to the rest of the stomodeal complex (Figure 4B, C, D).

The cells of the buccal sensory organs (also called pharyngeal organs [48]) develop next and will be associated with the future second commissure (Figures 3C and 4B, C, D, B′, C′, D′, B″, C″, D″). They arise as two pairs of clusters - one ventrolateral, followed by one dorsolateral - each consisting of three to five cells (asterisks in Figure 4B, C, D, B′, C′, D′, B″, C″, D″). The anteriormost (that is, first) commissure of the stomodeal complex is the third one to develop and comes from one pair of anterolateral clusters and an unpaired median cluster of cells that are already present early in development (Figure 4A, C, D). Finally, the posteriormost commissure forms late in development, after all major brain structures have been established (Figure 4D).

In addition to the four ring-like commissures of the stomodeal complex, a prominent circumbuccal nerve ring is positioned anterior to the brain (Figures 2E and 3E). The circumbuccal nerve ring innervates the circumoral sensory field, although these tracts do not appear until late in development (Figures 2D, E, 3D, E, 4D, and Additional file 4). The ring receives fibers from cells positioned within both the brain and the first trunk ganglion, the latter via a pair of inner connectives (Figures 2E and 3E).

### Neural development in the trunk

**Figure 7:**
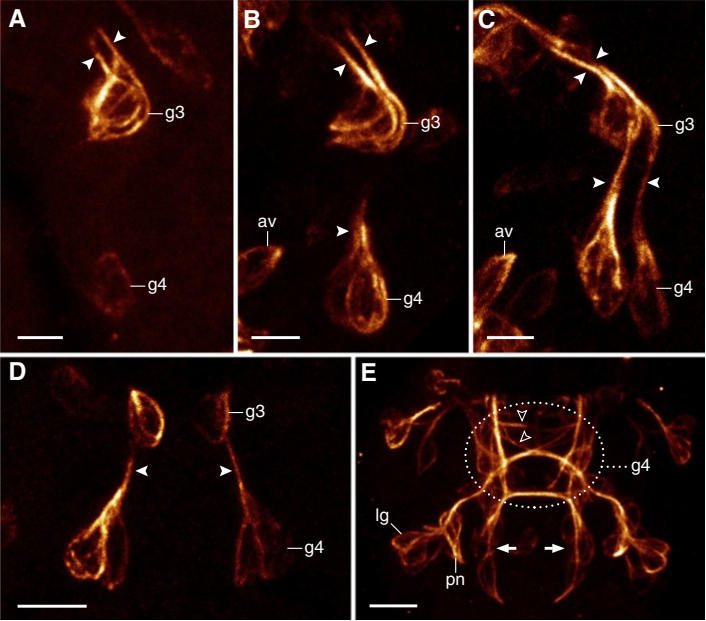
Development of the posterior two trunk ganglia in *Hypsibius dujardini*. Maximum CLSM projections of embryos labeled for acetylated α-tubulin in lateral view of successive stages (in (A-C)) and ventral view (in (D-E)). Solid arrowheads (in (A-D)) indicate longitudinal pioneering axons. Arrows (in (E)) point to neurons that may be associated with the hindgut or cloaca (cf. ref. [11]). **(A)** Development of the fourth trunk ganglion, which is delayed relative to that of the third trunk ganglion. **(B)** Beginning of pioneering axon growth in the fourth trunk ganglion anlage. Notice the proximity of the anteroventral cells to the fourth trunk ganglion due to the curved position of the embryo inside the egg. **(C)** Longitudinal pioneering axons (arrowheads) reach the anterior adjacent segment. **(D)** Detail of the developing central nervous system in the posterior region of an early-stage embryo. The fourth trunk hemiganglia anlagen are initially spaced far apart. **(E)** Late-stage embryo with peripheral nervous structures already present. The developing central fiber mass of the fourth trunk ganglion consists of several distinct commissures and fibers (open arrowheads). Abbreviations: av, anteroventral cells; lg, leg ganglion; g3 to g4, anlagen of trunk ganglia 3 to 4; pn, peripheral nerve. Scale bars: (A-C), 3 μm; (D), (E), 5 μm.

Although the neural structures of the head arise first in *H. dujardini*, the anlagen of the trunk ganglia can also be seen relatively early in development (Figures 2A, 5A, B, 6A, and 7A). They arise as a bilaterally symmetric pair of clusters in each trunk segment, with each cluster initially consisting of two to three cells (Figures 6A, B, C and 7A, B, C, D). Their development is characterized by an anterior-to-posterior progression, with anterior ganglia being further advanced than the posterior ones (Figures 2A, B, C, D, E, 5A, B, C, and 6A, B, C, D, E, F). All ganglia share a similar pattern of axon growth, in that two groups of pioneering axons grow anteriorly from each hemiganglion; one links to the hemiganglion of the anterior adjacent segment while the other grows contralaterally into a commissure that will later become part of the central fiber mass (Figures 6B, C, D and 7A, B, C, D, E). Although the anterior neurite begins developing first, both connections are actually formed simultaneously (hollow and solid arrowheads in Figure 6C, D). After a commissure is formed between the hemiganglia, more fibers then grow contralaterally into the anterior segment (Figure 6E). Later in development, this commissure becomes a part of the developing central fiber mass, which consists of several distinct commissures in addition to other fibers that do not follow specific pathways (Figures 6F and 7E).

Development of the first and fourth trunk ganglia of *H. dujardini* deviates from that of the second and third ganglia. The first ganglion shares two pairs of neural connections with the brain - the inner and outer connectives - that are both present in late-stage embryos (Figures 2E, 3E, and 5E). A pair of small clusters of cells is visible early in development directly ventral to the central commissure of the first ganglion and appears to be associated with the AV cells (asterisks in Figure 6A, B). The fourth trunk ganglion, on the other hand, lags behind in development, and the two early anlagen of the hemiganglia are further apart compared to the other three trunk ganglia (Figures 5A, B and 7A, D). Its presumptive central fiber mass consists of at least three prominent, distinct commissures in the late-stage embryo (Figure 7E). The middle commissure is associated with the anterior leg nerve and receives fibers from its respective ganglion (Figure 7E).

The peripheral nervous system of the trunk forms late in development, after the neural structures of the head have formed, and the trunk ganglia have become well established (Figures 2D, E and 5E). One of the most prominent peripheral nerves is a dorsolateral longitudinal nerve that spans the length of the body from anterior to posterior. While this nerve is present as a bilaterally symmetric pair in the trunk, it originates from the brain in a medial position (Additional file 5). In the second, third, and fourth trunk segments, this dorsolateral longitudinal nerve is connected to each hemiganglion via a branched anterior peripheral nerve (Additional file 6). In the first trunk segment, this connection is to the outer connective rather than to the first trunk ganglion (Additional file 6).

Each trunk ganglion is associated with two leg nerves that follow a strictly metameric pattern (Additional file 7). The anterior leg nerve is associated with the leg ganglion, the anlage of which consists of pioneering neurons that grow toward their respective trunk ganglion (Figures 2E and 7E). After this connection is made, neural tracts begin to grow from the trunk ganglion in the opposite direction, forming the posterior leg nerve, which displays a much weaker signal than the anterior leg nerve (Additional file 7). In addition to a pair of peripheral nerves and two pairs of leg nerves, the fourth trunk ganglion receives fibers from paired clusters of bipolar posterior neurons that might be associated with the hindgut or the cloaca (arrows in Figure 7E).

## Discussion

### No developmental evidence for a multisegmented tardigrade brain

Initial proposals of a multisegmented brain in tardigrades were primarily based on the trilobate arrangement of the adult brain and the innervation patterns of various heterotardigrade cephalic appendages [3,49,50]. Persson *et al*. [24] also described three brain segments in the eutardigrade *Halobiotus crispae* by homologizing the inner and outer lobes with the proto- and deutocerebrum, respectively, and a putative ‘subpharyngeal’ ganglion with the tritocerebrum. However, Zantke *et al*. [26] correctly point out that brain lobes do not necessarily correspond to brain segments and that cephalic appendages are highly diverse and likewise do not indicate segmental identity. On the contrary, studies on adult tardigrades and developing embryos support a one-segment head based both on morphology, for example, the position of the stomatogastric ganglion [23], as well as on Pax3/7 and Engrailed protein expression data, respectively [46]. The stomatogastric ganglion is associated with the second leg-bearing segment in the tardigrade *Macrobiotus* cf. *harmsworthi* and the tritocerebral segment in crustaceans, hexapods, and myriapods [23,51]. If this structure is homologous in these groups, it would indicate only two segments anterior to it in tardigrades, requiring the head to consist of a single segment [23]. Likewise, there are no additional expression domains of Pax3/7 and Engrailed in the anterior body region of the tardigrade embryo that would suggest multiple cephalic segments [46].

Our discovery of a single central brain commissure - the anlage of the central brain neuropil - throughout development in *H. dujardini* adds support for a one-segment tardigrade brain. This is in line with the single-segment hypothesis of the tardigrade head [23]. The fact that the brain cells arise in a dorsolateral position and increase in density from dorsolateral to dorsal throughout development suggests that the two outer and two inner brain lobes previously identified in *H. crispae* [24] develop from a single structure. This structure likely corresponds to the arthropod protocerebrum because its commissures grow exclusively dorsally over the mouth (that is, ‘preorally’ , as *H. dujardini* has a terminal mouth). The arthropod deutocerebrum, on the contrary, gives rise to both preoral and postoral fibers [14,52,53]. We could not identify such a deutocerebrum-like structure in embryos of *H. dujardini*.

Likewise, the lack of any additional cerebral ganglion anlagen during development further challenges the existence of a putative ‘subpharyngeal ganglion’ [24,50,54], as suggested in several studies [11,22,23,26,28,29]. It is possible that the evidence in favor of this structure was based on the AV cells described herein, which we have shown do not coalesce into a ganglion. In any case, these cells cannot be interpreted as part of the central nervous system because they lie outside it and display a morphology characteristic of peripheral sensory neurons. Therefore, our results do not support a multisegmented brain in the tardigrade *H. dujardini*, although gene expression studies, in particular of Hox genes [55-57], are required to confirm this hypothesis. Consequently, if the first trunk ganglion is in fact homologous to the arthropod deutocerebrum, as previously suggested [23], then the tritocerebrum must be an arthropod autapomorphy, as it is not differentiated as part of the brain in onychophorans either [34].

### The circumoral/circumbuccal nerve rings of arthropods and tardigrades are not homologous

**Figure 8:**
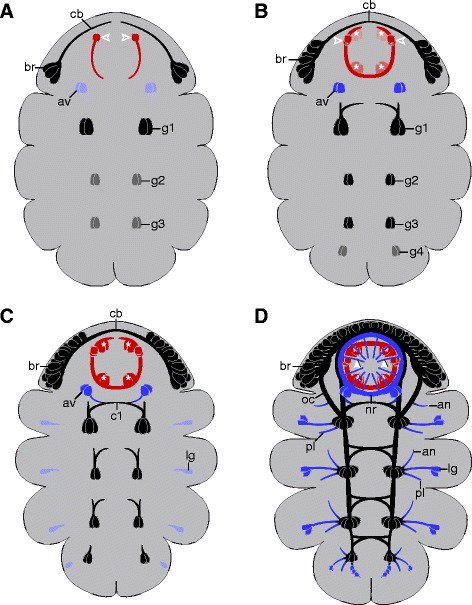
Simplified diagram of neural development in *Hypsibius dujardini*. Ventral view; anterior is up in all images. Hollow arrowheads indicate first neurons of the stomodeal nervous system (red). Solid arrowheads point to neurites innervating the circumoral sensory field. Asterisks indicate buccal sensory organs. Black, central nervous system; blue, peripheral nervous system. Developmental series, from (A-D). Abbreviations: an, anterior peripheral nerve; av, anteroventral cells; br, brain cells; c1, central commissure of the first trunk ganglion; cb, developing central brain neuropil (arising from a single commissure); g1 to g4, anlagen of trunk ganglia 1 to 4; lg, leg ganglion; nr, circumbuccal nerve ring; oc, outer connective; pl, posterior leg nerve.

An embryonic ‘circumoral nerve ring’ has been described from various arthropods, where it consists of the three segmental regions that constitute the arthropod brain: the proto-, deuto-, and tritocerebrum [14,51,53]. Tardigrades also have a nerve ring that encircles the mouth opening and persists in adults [23,24,26]. However, despite the superficial morphological resemblance between these structures in tardigrades and arthropods, they are unlikely to be homologous due to the lack of correspondences in the timing of development and innervation patterns. The circumbuccal nerve ring in *H. dujardini* does not arise until late in development, after the segmental ganglia have formed, and is a composite structure consisting of various neural tracts (Figure 8A, B, C, D). These include the inner connectives and the many neurites that innervate the circumoral sensory field and other peripheral structures (cf. [11]). On the other hand, the circumoral nerve ring of arthropods is not a separate structure, but rather a term describing the shape of the ganglia, commissures, and connectives exclusive to the three brain segments [14]. These three brain neuromeres are always among the first neural structures to appear and are located in the same position relative to the mouth across the arthropods [14,53,58]. We found no evidence for these neuromeres in embryos of *H. dujardini* and therefore suggest that the tardigrade circumbuccal nerve ring, which is separate from the brain, is an autapomorphy of the group.

### Support for the homology of segmental ganglia in arthropods and tardigrades

Previous studies of the adult nervous system in several tardigrade species have been quick to point out the resemblance between the nervous system in tardigrades and arthropods, namely the ‘rope ladder-like’ structure of the ventral nerve cords [11,24,28,29]. This term describes a nervous system consisting of bilaterally symmetric, segmental pairs of hemiganglia connected longitudinally by somata-free connectives and transversely by commissures [59]. A similar arrangement has been described from some annelid taxa [60], but the prevalent hypothesis is that their segmentation, including segmental ganglia, evolved independently from that of arthropods ([17,60-62]; but see [63,64] for an opposing view). Although Zantke *et al*. [26] could not detect transverse fibers between each pair of hemiganglia in the tardigrade *Macrobiotus hufelandi*, such fibers have been described in virtually every other investigation of tardigrade neuroanatomy [11,20,24,28,29,40]. Consequently, Mayer *et al*. [11] proposed a hypothesis homologizing the segmental ganglia in tardigrades and arthropods based on these and other features, for example, segmentally repeated sets of neurons and an anteriorly shifted position of ganglia within each segment.

Our investigation of *H. dujardini* supports this hypothesis by revealing several developmental features of the ganglia that are also conserved in many arthropod taxa (reviewed in refs [65-68]). Segmental trunk ganglia in *H. dujardini* arise as bilaterally symmetric hemiganglia that are initially positioned far apart within each segment. Examinations of numerous embryos revealed that the anlagen of the hemiganglia arise in the same position, with a similar number of cells, and in the same sequence in every specimen, suggesting that the trunk ganglia of tardigrades have individually identifiable neurons. This also holds true for arthropods, especially crustaceans and insects, where individual neurons have in fact been categorized in several taxa [66,67,69,70]. More similarities are seen regarding the neural pathways; for example, each hemiganglion projects pioneering axons from its dorsal side and the longitudinal connective arises simultaneously with the transverse commissure in both tardigrades and arthropods [58]. In summary, these developmental characters add to previous support [11] for a single origin of segmental ganglia within the Panarthropoda, irrespective of whether or not they were present in the onychophoran lineage. This finding clearly speaks against the sister group relationship of tardigrades with nematodes [6,9,71].

### Issues regarding the reconstruction of the last common ancestor of Panarthropoda

Several developmental features that were characteristic of arthropods are revealed herein to be present in tardigrades as well. These characters include i) a ‘rope ladder-like’ nervous system with segmental ganglia and somata-free connectives [14,72], ii) individually identifiable, segmentally repeated sets of neurons [70,73], iii) simultaneous development of longitudinal and transverse pioneering axons in the trunk [58], iv) contralateral fibers linking adjacent segments [70,73], and v) initial appearance of leg nerves in an intermediate position along the proximodistal axis within each leg ([58]; Figure 8C). These features most likely evolved in the tardigrade/arthropod lineage or, alternatively, were lost in onychophorans.

On the other hand, the pattern of neural development in tardigrades is also remarkably similar to that in onychophorans despite, for example, the absence of ring commissures in tardigrades [11,24] and segmental ganglia in onychophorans [17,19]. These two groups share several features to the exclusion of arthropods, namely i) all brain neuropils arising from a single, anteriormost/protocerebral commissure [34], ii) lack of posterior-growing pioneering axons (that is, longitudinal pioneering axons grow only in the anterior direction) [17], iii) development of lateral (that is, leg and peripheral) nerves only after the ventral nerve cords have been established [17,19], and iv) development of the anterior leg nerve followed by the posterior one [17]. These features are either symplesiomorphies of onychophorans and tardigrades that have been inherited from the last common ancestor of Panarthropoda or synapomorphies uniting these two groups - a relationship that has in fact been suggested previously [12].

Unfortunately, molecular analyses have thus far failed to provide an unambiguous panarthropod phylogeny, owing to the unstable position of tardigrades [74]. Nearly all molecular studies place the onychophorans as the sister group of the arthropods [9,10,71,74,75]; however, the tardigrades are commonly placed as sister either to the Onychophora [12], the Onychophora + Arthropoda [10,75], or to the Nematoda [9,71,76]. Although the sister group relationship of tardigrades with nematodes is often dismissed due to a long-branch attraction artifact [10], other topologies are highly dependent upon the choice of substitution models [10,12,74]. A study using microRNA data [10] claimed to ‘resolve’ panarthropod relationships based on a single shared copy, but a recent analysis [77] has questioned the utility of microRNA data for phylogenetic inference. In short, it becomes clear that the position of tardigrades, and the issue of panarthropod phylogeny in general, remains an open question, making it difficult to reconstruct the last common ancestor of Panarthropoda.

Interestingly, none of the topologies based on molecular data supports the sister-group relationship of tardigrades with arthropods, which is supported by several morphological studies [11,78,79]. However, the lack of molecular evidence for this relationship might be explained by another type of long-branch attraction artifact, one that causes slippage of a branch - in this case, the Tardigrada - down the tree due to signal erosion [80]. In any case, accepting the monophyly of Panarthropoda [3,75] would place the tardigrades as sister to either Arthropoda [11,78,79] or to Onychophora + Arthropoda [10,75,81] as the two most plausible hypotheses. If tardigrades are sister to the arthropods to the exclusion of onychophorans, the last common ancestor of Panarthropoda may have had widely spaced, medullary ventral nerve cords established by anteriorly growing pioneering axons. A pair of leg nerves may have been present that developed after the longitudinal tracts had been established in the embryo, with the anterior leg nerve developing first.

Alternatively, if the onychophorans are the sister group of the arthropods to the exclusion of tardigrades, then the panarthropod ancestor may have had a ‘rope ladder-like’ nervous system, including anteriorly shifted, segmental ganglia fused at the midline, with somata-free connectives and contralateral, intersegmental commissures. The leg nerves may have developed from an intermediate proximodistal position within each leg and may have been associated with an additional peripheral ganglion. However, since onychophorans, and not tardigrades, share the most nervous system characters with other protostomes - cycloneuralians lack somata-free segmental ganglia [82], while an orthogonal nervous system may have been present in the last common ancestor of protostomes [15,83] - we find the second scenario to be unlikely. Therefore, based on our neural developmental data, we favor the first alternative, that is, the sister group relationship of tardigrades and arthropods, as the most parsimonious hypothesis describing the evolution of neural development in panarthropods.

## Conclusions

The present study revealed that tardigrade neural development, while also sharing several features with onychophorans [17], closely resembles that of arthropods [58,70,73]. Thus, the hypothesis suggesting that tardigrades are sister to nematodes, based on some molecular studies [6,7,9], is incompatible with our neuroanatomical data, which supports previous claims that such a placement results from analysis artifacts, more specifically long-branch attraction [10,11]. Our data further show that the tardigrade circumbuccal nerve ring has a unique structure and is an autapomorphy of the group, while the brain develops in a bilaterally symmetric pattern, similar to that of onychophorans and arthropods. Neither of these structures is comparable to the collar-shaped brain of cycloneuralians (Nematoida + Scalidophora) [3,82,84], reinforcing it as a defining feature of the Cycloneuralia [85] rather than a synapomorphy uniting the tardigrades with nematodes.

## Additional files

Additional file 1:
**Overview of** ***Hypsibius dujardini*** **embryo.** CLSM projections of double-labeled stage 16 embryo (*sensu* Gabriel *et al.* [42]) in lateral view. Anterior is right in all images. **A**. DNA labeling. **B**. Anti-acetylated α-tubulin labeling. Arrowheads indicate the ventral nerve cords. **C**. Merged image showing both markers. Abbreviation: br, brain. Scale bars: A-C, 10 μm. Additional file 2:
**Nervous system of a late-stage embryo of** ***Hypsibius dujardini*** **.** Anti-acetylated α-tubulin immunolabeling, rocking animation, MP4 file. Anterior is up. Additional file 3:
**Nervous system of a late-stage embryo of** ***Hypsibius dujardini*** **.** Anti-acetylated α-tubulin immunolabeling, still frame from the animation in Additional file 1. Anterior is up. Arrows indicate apical neurites of the anteroventral cells growing toward the body surface. Scale bar: 10 μm. Additional file 4:
**Nervous system of a late-stage embryo of** ***Hypsibius dujardini*** **.** Anti-acetylated α-tubulin immunolabeling, ventral view. Anterior is up. Confocal z-series micrograph showing the ventralmost part of the nervous system, including the circumbuccal nerve ring and the neurites of the circumoral sensory field (arrows). Abbreviation: nr, circumbuccal nerve ring. Scale bar: 5 μm. Additional file 5:
**Detail of the head region of a late-stage embryo of** ***Hypsibius dujardini*** **.** Anti-acetylated α-tubulin immunolabeling, confocal z-series projections. Optical sections from a single stack from ventral (in A) to dorsal (in C). Anterior is up in all images. Arrowheads (in C) point to neurons of the stomodeal nervous system. Asterisks indicate buccal sensory organs. Note that the dorsomedian longitudinal nerve originates in the central brain neuropil and splits into a paired structure (that is, the dorsolateral nerves) further posteriorly. Abbreviations: br, brain cells; cb, developing central brain neuropil; dn, dorsomedian longitudinal nerve; ic, inner connectives; oc, outer connectives. Scale bars: A-C, 5 μm. Additional file 6:
**Anterior peripheral nerves of a late-stage embryo of** ***Hypsibius dujardini*** **.** Anti-acetylated α-tubulin immunolabeling, ventrolateral view. Anterior is left. Arrows indicate points of origin of the anterior peripheral nerve in each segment. **A**. Three-dimensional projection (requires red-cyan 3D glasses). **B**. Maximum CLSM projection. Scale bars: A, B, 5 μm. Additional file 7:
**Leg nerves of a late-stage embryo of** ***Hypsibius dujardini*** **.** Anti-acetylated α-tubulin immunolabeling, ventrolateral view. Anterior is left. Arrows indicate anterior (left) and posterior (right) leg nerves associated with the first trunk ganglion. **A**. Three-dimensional projection (requires red-cyan 3D glasses). **B**. Maximum CLSM projection. Scale bars: A, B, 5 μm.

## Abbreviations

A: anterior
an: anterior peripheral nerve
av: anteroventral cells
bo: buccal sensory organs
br: brain cells
c1: c2, commissures of trunk ganglia 1 and 2
cb: developing central brain neuropil
CLSM: confocal laser scanning microscopy
D: dorsal
dn: dorsomedian longitudinal nerve
g1 to g4: anlagen of trunk ganglia 1 to 4
go: gonad anlage
ic: inner connective
lg: leg ganglion
M: median
NGS: normal goat serum
nr: circumbuccal nerve ring
oc: outer connective
P: posterior
PBT: phosphate-buffered saline with 1% Triton X-100
pd: posterodorsal cells
pl: posterior leg nerve
pn: peripheral nerve
V: ventral
