# Supplementary figures and images for: Neural development in the tardigrade Hypsibius dujardini based on anti-acetylated α-tubulin immunolabeling
Source: EvoDevo. 2015 Apr 25;6:12. doi: 10.1186/s13227-015-0008-4 (PMC4458024; doi:10.1186/s13227-015-0008-4)

A

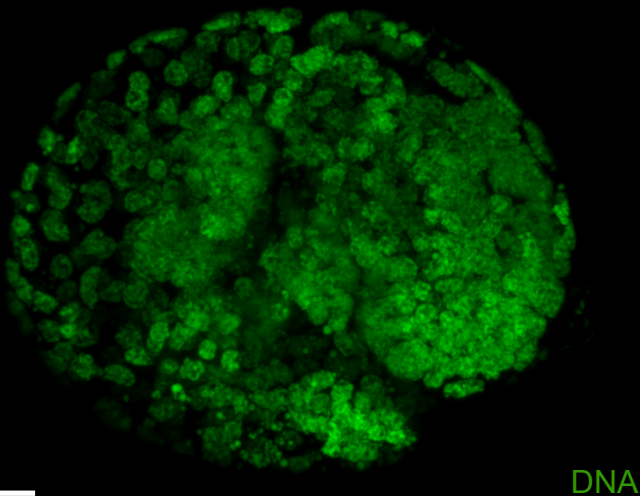

DNA

B

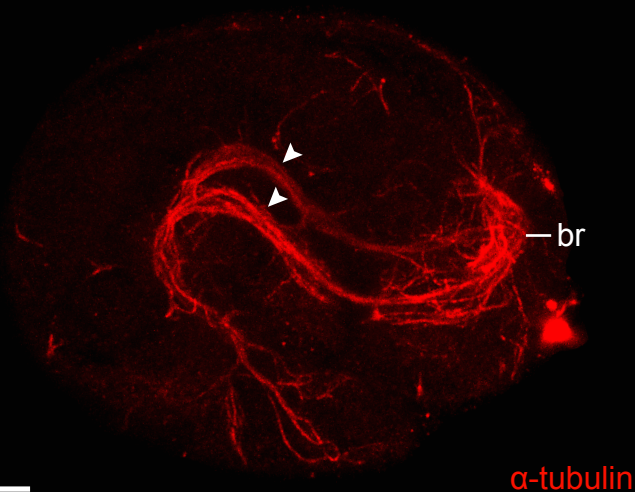 $\alpha$ -tubulin

C

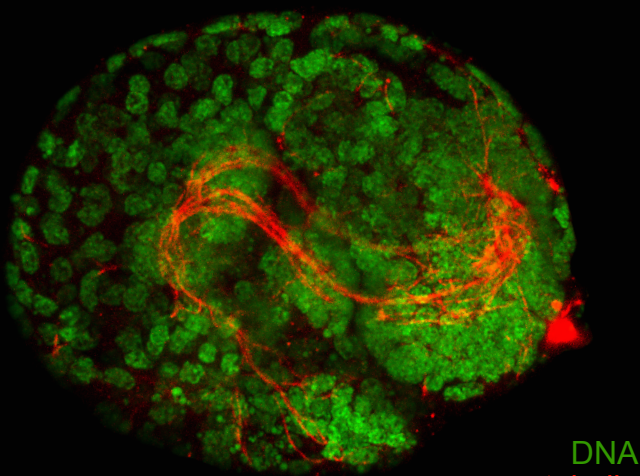DNA  
 $\alpha$ -tubulin

Supplement: Additional file 1: — Overview of Hypsibius dujardini embryo. CLSM projections of double-labeled stage 16 embryo (sensu Gabriel et al. [42]) in lateral view. Anterior is right in all images. A. DNA labeling. B. Anti-acetylated α-tubulin labeling. Arrowheads indicate the ventral nerve cords. C. Merged image showing both markers. Abbreviation: br, brain. Scale bars: A-C, 10 μm. [file 13227_2015_8_MOESM1_ESM.pdf]

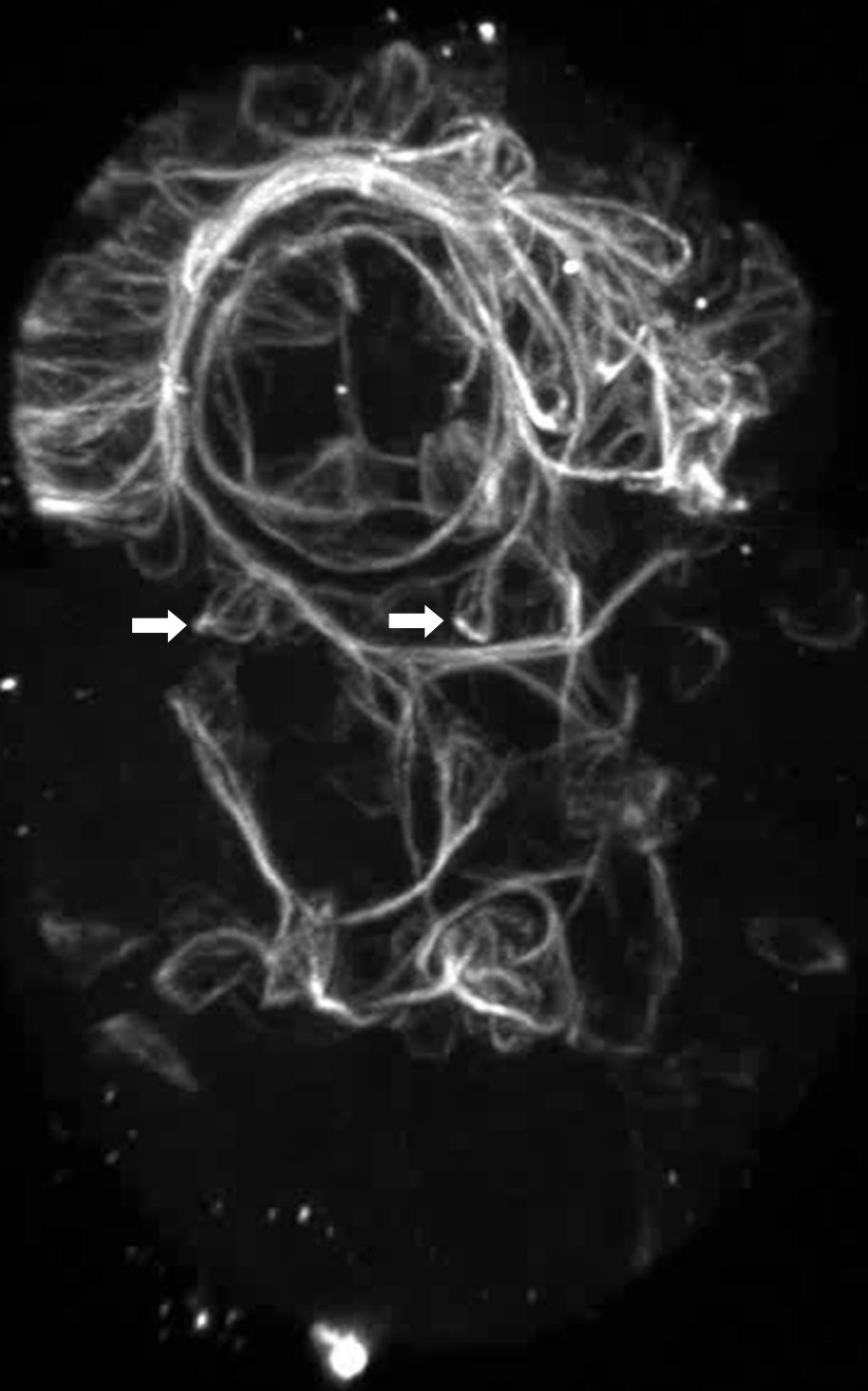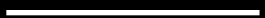

Supplement: Additional file 3: — Nervous system of a late-stage embryo of Hypsibius dujardini . Anti-acetylated α-tubulin immunolabeling, still frame from the animation in Additional file 1. Anterior is up. Arrows indicate apical neurites of the anteroventral cells growing toward the body surface. Scale bar: 10 μm. [file 13227_2015_8_MOESM3_ESM.pdf]

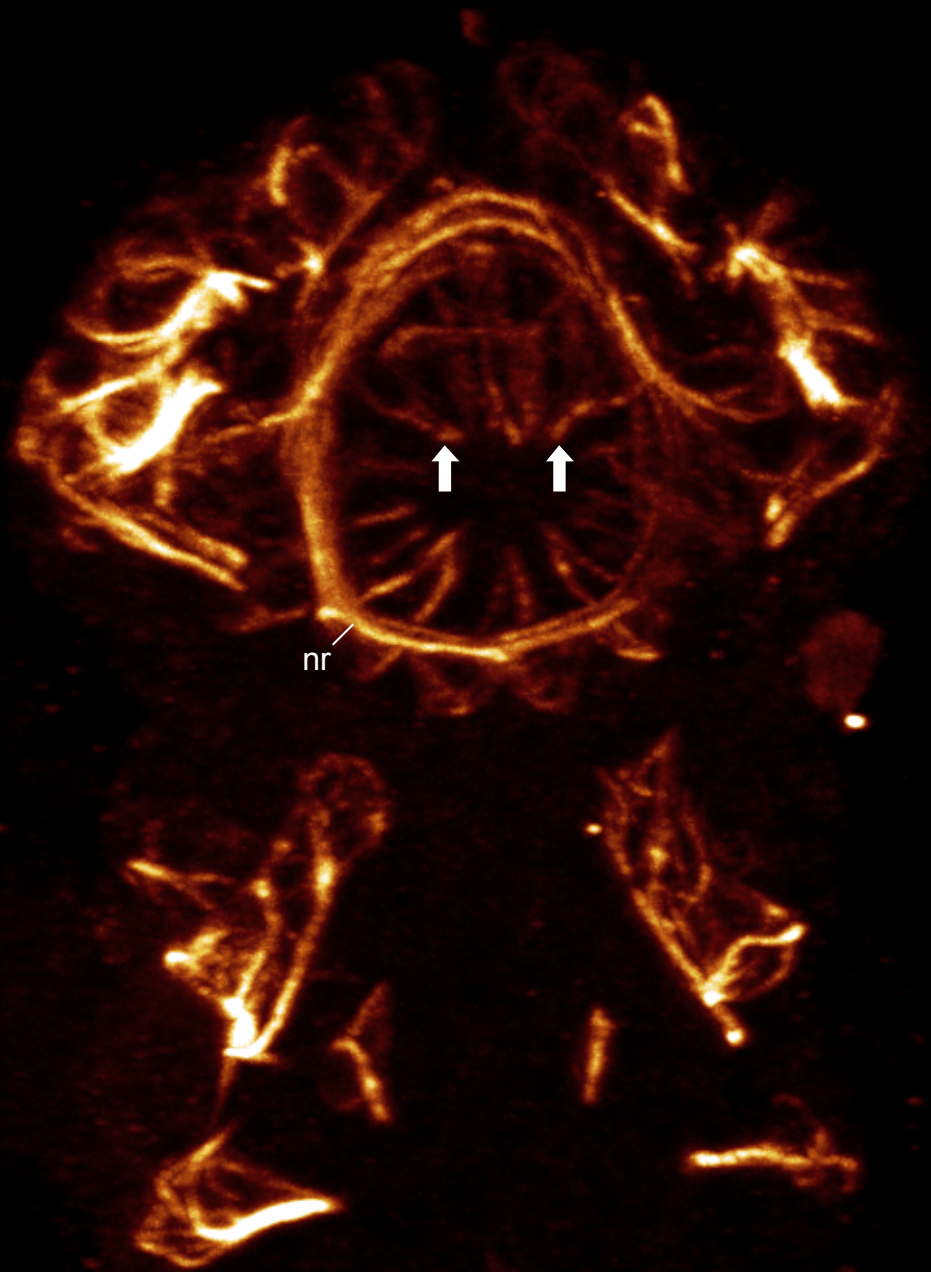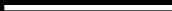

Supplement: Additional file 4: — Nervous system of a late-stage embryo of Hypsibius dujardini . Anti-acetylated α-tubulin immunolabeling, ventral view. Anterior is up. Confocal z-series micrograph showing the ventralmost part of the nervous system, including the circumbuccal nerve ring and the neurites of the circumoral sensory field (arrows). Abbreviation: nr, circumbuccal nerve ring. Scale bar: 5 μm. [file 13227_2015_8_MOESM4_ESM.pdf]

A

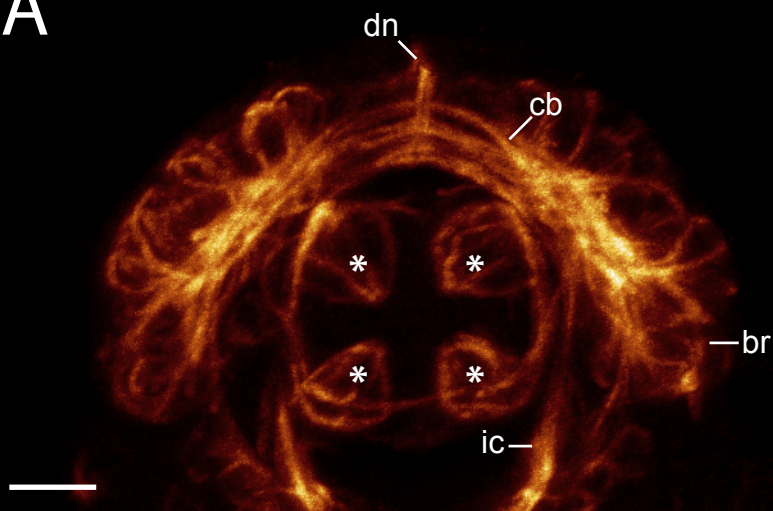

B

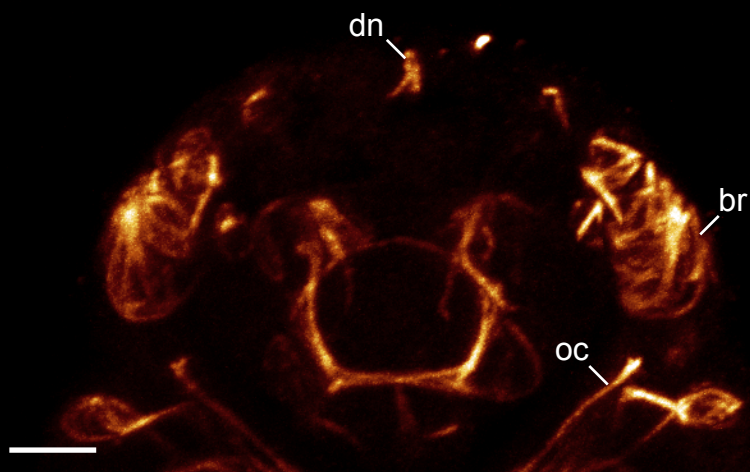

C

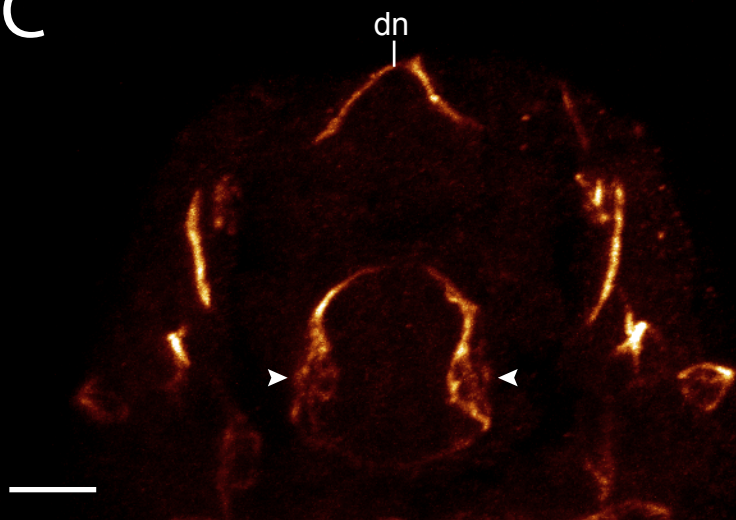

Supplement: Additional file 5: — Detail of the head region of a late-stage embryo of Hypsibius dujardini . Anti-acetylated α-tubulin immunolabeling, confocal z-series projections. Optical sections from a single stack from ventral (in A) to dorsal (in C). Anterior is up in all images. Arrowheads (in C) point to neurons of the stomodeal nervous system. Asterisks indicate buccal sensory organs. Note that the dorsomedian longitudinal nerve originates in the central brain neuropil and splits into a paired structure (that is, the dorsolateral nerves) further posteriorly. Abbreviations: br, brain cells; cb, developing central brain neuropil; dn, dorsomedian longitudinal nerve; ic, inner connectives; oc, outer connectives. Scale bars: A-C, 5 μm. [file 13227_2015_8_MOESM5_ESM.pdf]

A

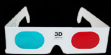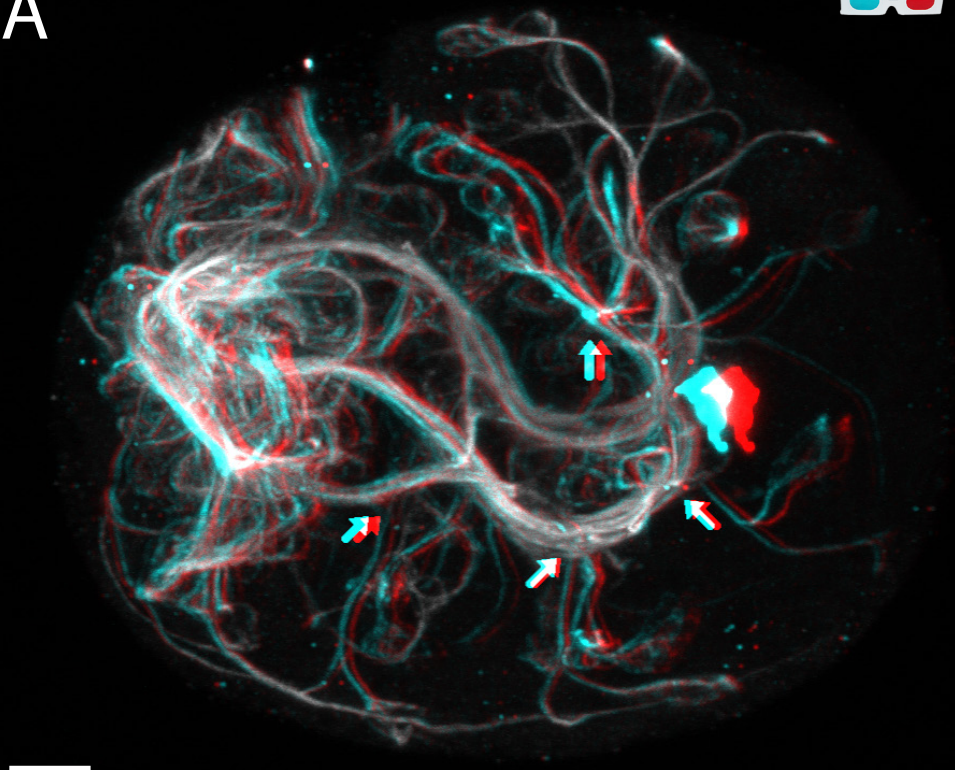

B

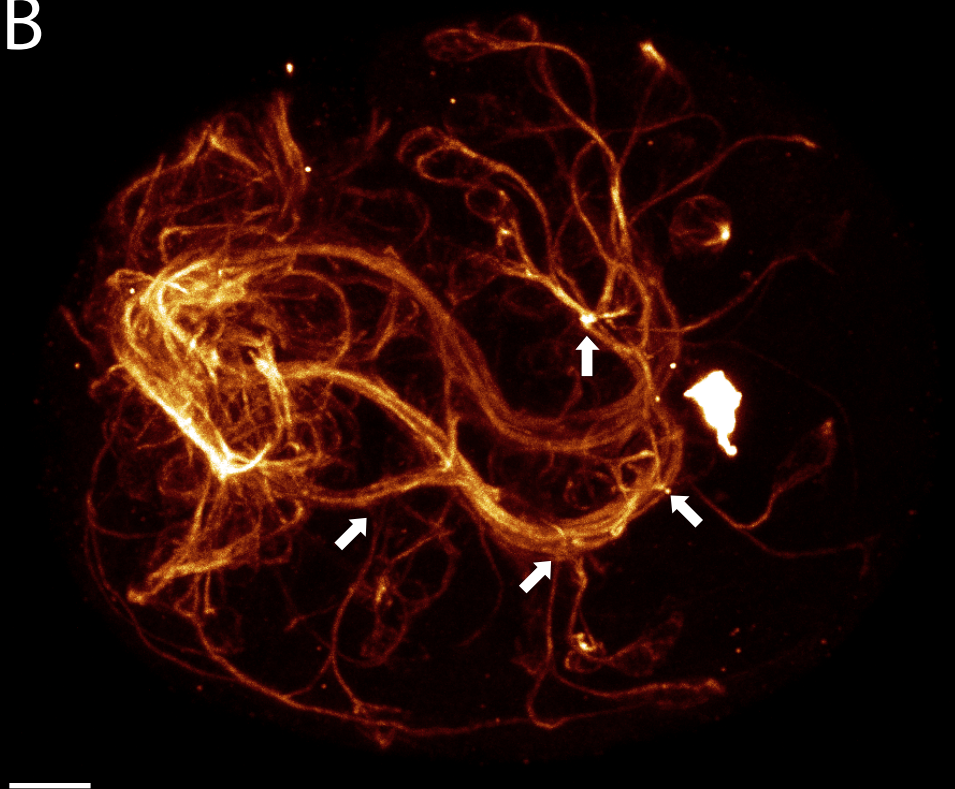

Supplement: Additional file 6: — Anterior peripheral nerves of a late-stage embryo of Hypsibius dujardini . Anti-acetylated α-tubulin immunolabeling, ventrolateral view. Anterior is left. Arrows indicate points of origin of the anterior peripheral nerve in each segment. A. Three-dimensional projection (requires red-cyan 3D glasses). B. Maximum CLSM projection. Scale bars: A, B, 5 μm. [file 13227_2015_8_MOESM6_ESM.pdf]

A

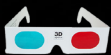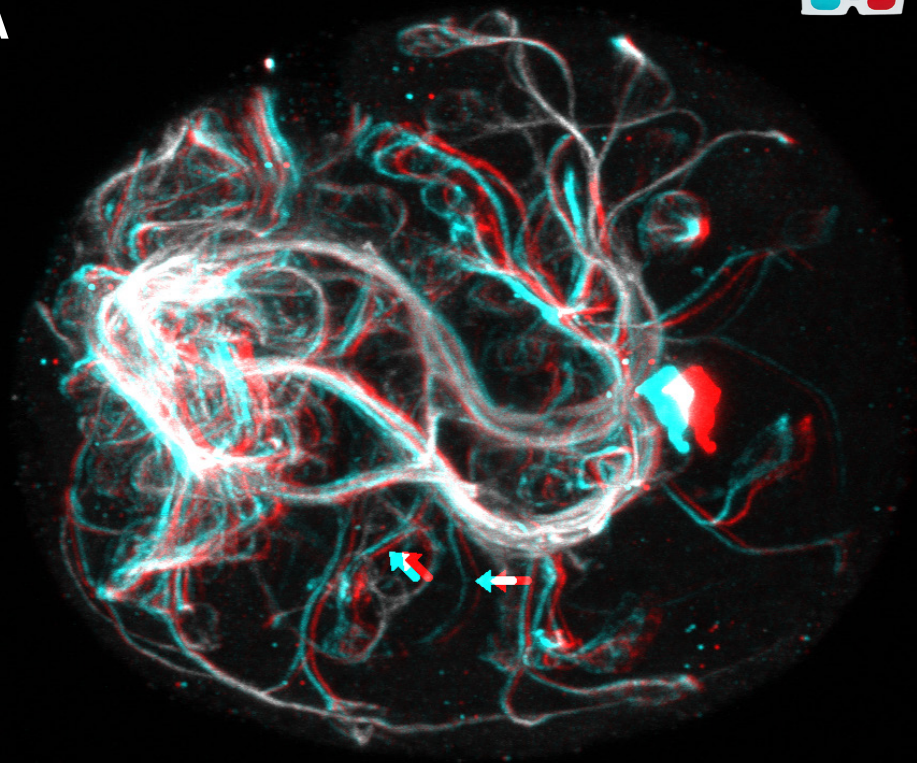

B

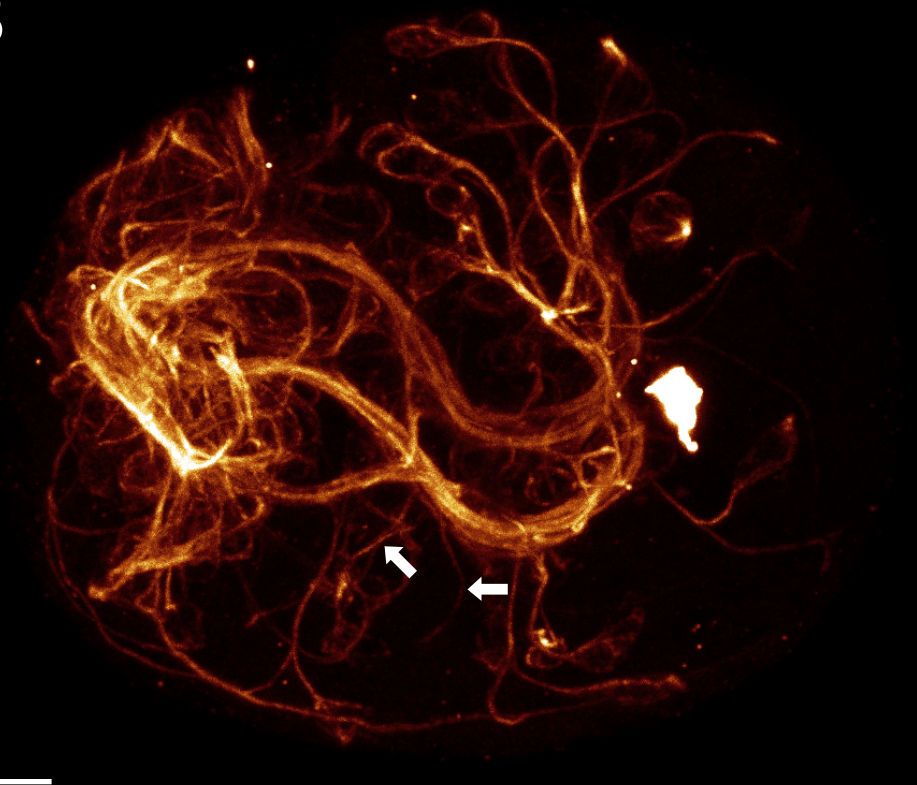

Supplement: Additional file 7: — Leg nerves of a late-stage embryo of Hypsibius dujardini . Anti-acetylated α-tubulin immunolabeling, ventrolateral view. Anterior is left. Arrows indicate anterior (left) and posterior (right) leg nerves associated with the first trunk ganglion. A. Three-dimensional projection (requires red-cyan 3D glasses). B. Maximum CLSM projection. Scale bars: A, B, 5 μm. [file 13227_2015_8_MOESM7_ESM.pdf]
